# Targeting the complement system for the management of retinal inflammatory and degenerative diseases

**DOI:** 10.1016/j.ejphar.2016.03.001

**Published:** 2016-09-15

**Authors:** Heping Xu, Mei Chen

**Affiliations:** Centre for Experimental Medicine, School of Medicine, Dentistry & Biomedical Sciences, Queen's University Belfast, UK

**Keywords:** Age-related macular degeneration, Diabetic retinopathy, Glaucoma, Uveoretinitis, Inflammation, Complement, Treatment

## Abstract

The retina, an immune privileged tissue, has specialized immune defense mechanisms against noxious insults that may exist in diseases such as age-related macular degeneration (AMD), diabetic retinopathy (DR), uveoretinitis and glaucoma. The defense system consists of retinal innate immune cells (including microglia, perivascular macrophages, and a small population of dendritic cells) and the complement system. Under normal aging conditions, retinal innate immune cells and the complement system undergo a low-grade activation (parainflammation) which is important for retinal homeostasis. In disease states such as AMD and DR, the parainflammatory response is dysregulated and develops into detrimental chronic inflammation. Complement activation in the retina is an important part of chronic inflammation and may contribute to retinal pathology in these disease states. Here, we review the evidence that supports the role of uncontrolled or dysregulated complement activation in various retinal degenerative and angiogenic conditions. We also discuss current strategies that are used to develop complement-based therapies for retinal diseases such as AMD. The potential benefits of complement inhibition in DR, uveoretinitis and glaucoma are also discussed, as well as the need for further research to better understand the mechanisms of complement-mediated retinal damage in these disease states.

## 1 The retina – an immune privileged tissue

**Fig. 1:**
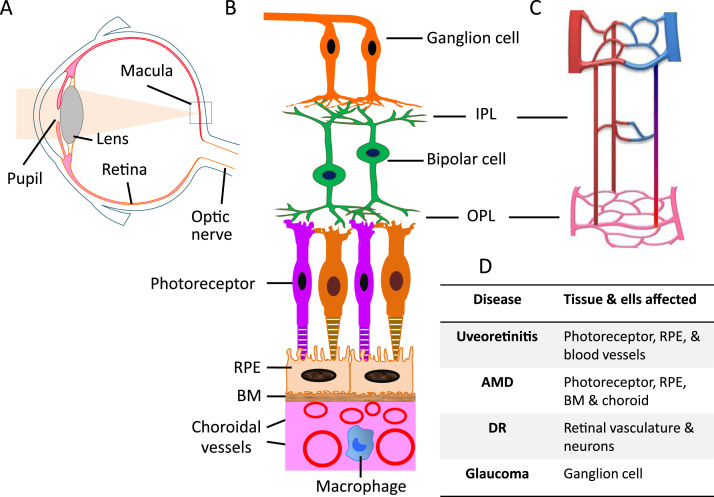
Retinal neuronal and vascular structure and retinal disease. A, diagram of a human eye. Light passes through the pupil and is focused by the lens onto the macula of the retinal layer at the back of the eye. B, the retina consists of three layers of neurons, photoreceptor, bipolar, and ganglion cells. The RPE monolayer together with Bruch's membrane (BM) form the outer blood retinal barrier that separates the neuroretina from the choroid. Choroidal circulation provides oxygen and nutrients to the outer retina. C, the retina has an interconnected network of three vascular layers located in the ganglion cell/nerve fibre layer, inner plexiform layer (IPL), and outer plexiform layer (OPL). D, retinal tissue and cells that are affected under different disease conditions.

The retina is essential for perception of vision. Light enters the eye through the cornea and iris and passes through the vitreous body to project onto the retina where the light signal is converted into electric impulses (Fig. 1A). The visual cycle occurs at the interface between the retina and retinal pigment epithelium (RPE), leading to the depolarization of photoreceptors (the rods and the cones). The electrical impulses converge via bipolar cells and ganglion cells onto the optic nerve, and then into the visual cortex (Fig. 1B). The inner retinal cells obtain nutrients and oxygen from the retinal circulation (Fig. 1C), whereas the outer retinal layers (which consists of the outer nuclear layer (ONL), photoreceptor inner segment (IS), and outer segment (OS)) are avascularized and nutrients and oxygen are supplied by the choroidal circulation (Fig. 1B). To ensure good visual function, this complex and sophisticated structure must be maintained throughout life, and even a minor perturbation may cause devastating visual impairment.

The eye has special mechanisms to protect the retina from exogenous and endogenous insults, which not only reduces the risk of infection, but also prevents inappropriate immune responses, thereby reducing the risk of inflammation–mediated retinal damage. Firstly, the retina is protected by physical barriers. The blood retina barrier (BRB) is formed by tight junctions between vascular endothelial cells (inner BRB, iBRB) and RPE cells (outer BRB, oBRB), and ensures that pathogens, circulating cells and molecules do not freely pass into the retinal parenchyma. The BRB also sequesters retinal antigens within the intraocular compartment avoiding T cell activation, a phenomenon called immunological ignorance (Avichezer et al., 2003, Forrester et al., 2008, Forrester et al., 2010, Forrester and Xu, 2012). Secondly, the retina has no lymphatic system. Therefore, when the retina suffers from any insult, the endogenous alarmins are unlikely to be detected by circulating or choroidal antigen presenting cells (APCs) if the BRB is intact. Thirdly, the retina has a sophisticated immune regulatory system orchestrated by retinal cells, including various neurons and RPE cells (Streilein, 1999, Streilein et al., 2002, Wenkel and Streilein, 2000). These retinal cells express immune modulators that can suppress immune cell activation. Examples of the immune modulatory mechanisms include (but are not limited to) the CD200-CD200R (Dick et al., 2003) and CX3CL1-CX3CR1 (Combadiere et al., 2007) pathway, thrombospondin-1, TGF-β, CTLA4, CTLA2, and various complement inhibitors (Horie et al., 2010, Kawazoe et al., 2012, Mochizuki et al., 2013, Sugita et al., 2009, Zamiri et al., 2005). Mechanisms to induce the death of infiltrating immune cells through Fas ligand (FasL) and Tumor Necrosis Factor-related apoptosis-inducing ligand (TRAIL) pathways also exist in the retina (Ferguson and Griffith, 1997, Ferguson and Griffith, 2007, Kazama et al., 2008). Furthermore, ocular fluids contain immunoinhibitory molecules such as TGF-β2 and neuropeptides, including α-melanocyte-stimulating hormone and vasoactive intestinal peptide (Taylor, 1996, Taylor and Lee, 2010). The retina, therefore, represents an immune privileged tissue (Forrester et al., 2008, Gregerson, 1998, Streilein et al., 2002).

Despite being an immune privileged tissue, both retinal neurons and the vascular system may degenerate in conditions such as diabetic retinopathy (DR), age-related macular degeneration (AMD), uveoretinitis and glaucoma (Fig. 1D). Although the initial triggers of different diseases may differ, the retinal immune response following the initial insult shares many common pathways. Such inflammatory response secondary to retinal damage critically contributes to further neuronal and vascular degeneration in the aforementioned sight-threatening diseases. Here, we review the current knowledge on how the retinal innate immune system responds to insults with a strong focus on the complement system, and further discuss how the knowledge can be applied to developing complement based therapy.

## 2 Tissue insults, inflammation and disease – the concept

The central role of the immune system is to protect the host from exogenous and endogenous insults. When a tissue suffers from non-infectious noxious insults, a cell-autonomous response is initiated for stressor elimination or stress adaptation. This may include the upregulation of the autophagy pathway, the initiation of DNA repair, and the induction of chaperones to help to prevent protein misfolding (the adaptation response). The adaptation response is especially important for aging and diabetes. In addition, a non-cell-autonomous response may also be initiated wherein stressed cells release cytokines, chemokines and growth factors that affect neighbouring cells within the tissue, promoting tissue level adaptation. In the literature, this tissue autonomous response (cell-autonomous or non-cell-autonomous) is often called “inflammation” when inflammatory cytokines and chemokines are released by stressed cells.

Although many cells can detect tissue stress, this function is performed most efficiently by specialized sensory cells, such as tissue resident macrophages. Thus, pathogens and generic stressors such as hypoxia and oxidized lipids/proteins are primarily delegated to macrophages. Tissue macrophages may orchestrate tissue level defence and adaptations by releasing inflammatory mediators, including complement components. If the level of insult exceeds the repair capacity of tissue macrophages, they may recruit circulating immune cells to sites of damage. A low-level of tissue insult may initiate a protective para-inflammation (Chovatiya and Medzhitov, 2014, Medzhitov, 2008). When the stress persists for a sustained period of time, the affected tissue may mal-adapt leading to loss of function. In addition, the immune system may respond inappropriately to tissue stress due to genetic and epigenetic modifications resulting in dysregulated para-inflammation (chronic inflammation). Tissue pathology (disease) may occur as a result of tissue mal-adaptation or immune dysregulation.

## 3 Retinal innate immune defence and disease

As an immune privileged tissue, circulating immune cells are not able to enter the retina to deal with endogenous insults under normal physiological conditions. However, the retina has a unique immune defence system consisting of innate immune cells and the complement system. The retina contains at least three types of innate immune cells: microglia, perivascular macrophages, and dendritic cells (DCs) (Forrester et al., 2010, Xu et al., 2009). Although both perivascular macrophages and microglia express CD11b and F4/80, the former expresses high levels of CD14 (LPS receptor) and CD45, whereas microglia are CD14^-^CD45^low^ (Dick et al., 1995, Fischer and Reichmann, 2001, Tavazzi et al., 2014). Perivascular macrophages safeguard retinal vessels and are critically involved in retinal vascular homeostasis. Whether or not the retina has DCs has been a debate for decades. Early work by Zhang and colleagues reported a small population of MHC-II^+^ cells in rat retina (Zhang et al., 1997). Using flow cytometry analysis, Gregerson and Yang detected a small population of CD11c^+^ DEC205^+^ DCs in normal mouse retina (Gregerson and Yang, 2003). They further confirmed the existence of retinal CD11c cells using CD11c-DTR transgenic mice (Lehmann et al., 2010), although a later study suggested that rd8 mutation in the Crb1 gene may contribute to the abnormal number of CD11c^+^ cells in the retina in CD11c-eYFP transgenic mice (Chen et al., 2013b). Previously, we identified a small population of MHC-II^+^33D1^+^ DCs in mouse retina which are located around the optic disc and the peripheral retinal margin area (Xu et al., 2007). The function of these cells is unclear, but their strategic location suggests they may be “gatekeepers” of the retina. In experimental autoimmune uveoretinitis (EAU), these DCs are activated prior to overt retinal inflammation and their activation is associated with early cell infiltration around the optic disc and peripheral retinal margin (Xu et al., 2007). These retinal DCs are activated in experimental models of optic nerve degeneration and they can phagocytose dead ganglion cells (Heuss et al., 2014, Lehmann et al., 2010). Ganglion cell degeneration/optic disc cupping is the key biological change in glaucoma. The MHC-II^+^33D1^+^ juxtapapillary DCs may be critically involved in the pathogenesis of glaucoma. Microglia form an important part of retinal immune defence. They are located in the inner layers of the retina, and are distributed into three layers: the ganglion layer (GL), the inner plexiform layer (IPL) and outer plexiform layer (OPL) (Chen and Xu, 2015). Under normal physiological conditions, microglia are in a resting state with a small cell body and long, thin dendrites. They are activated in disease states, such as in AMD, DR, and uveoretinitis. Microglial activation may contribute to further retinal damage and disease progression. The role of retinal microglia in retinal health and disease has been reviewed extensively elsewhere (Karlstetter et al., 2010, Karlstetter et al., 2015, Langmann, 2007, Li et al., 2015).

In addition to retinal innate immune cells, compelling evidence suggests that the complement system also plays a critical role in protecting the retina from exogenous and endogenous insults. Dysregulated complement activation may contribute to retinal disease and targeting the complement system may offer opportunities for therapy.

## 4 The complement system

**Fig. 2:**
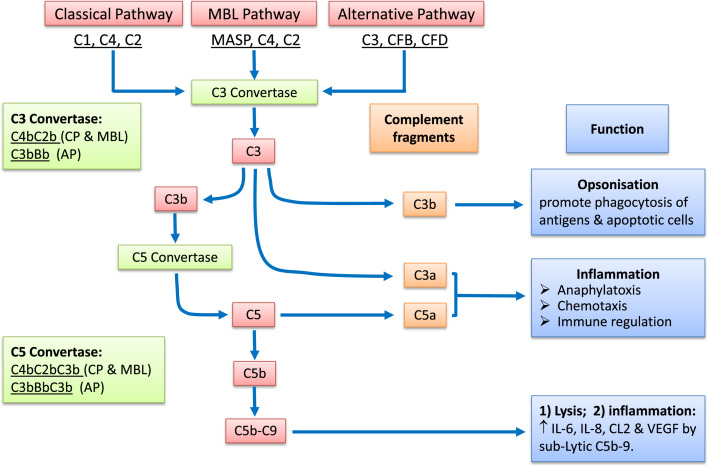
Complement activation and immune regulation. The complement system can be activated by the classical pathway (CP), mannose-binding lectin (MBL) pathway and the alternative pathway (AP); all leads to the cleavage of C3 and C5 and the formation of C5b-C9. C3 is cleavaged by C3 convertases, which include C4bC2b of the CP and MBL pathways and C3bBb of the AP. C5 is cleavaged by C5 convertases, which include C4bC2bC3b of the CP and MBL pathways and C3bBbC3b of the AP. Activation of the complement system generates C4a, C3a, C3b and C5a fragments that are actively involved in immune responses. C3b opsonizes foreign antigens and apoptotic cells, promoting phagocytosis. C3a and C5a are anaphylatoxins that have multiple immune regulatory roles. The C5b-C9 may directly kill pathogens or cells. The sub-lytic C5b-9 can also promote inflammation.

The complement system is an important part of the innate immune system. It complements antibodies and phagocytes to clear pathogens from the host. The complement system consists of over 30 small proteins and protein fragments and the majority of them are thought to be produced in the liver as inactive precursors and released into the circulation for tissue distribution. The complement system can be activated by at least three pathways: the classical pathway (CP), the mannose-binding lectin (MBL) pathway, and the alternative pathway (AP) (Fig. 2). There are two critical steps for the full activation of the complement pathways: C3 cleavage and C5 cleavage. A fully activated complement system results in the formation of the membrane attack complex (MAC, or C5b-C9) (Fig. 2) that can kill pathogens and cells. It is important to note that under physiological conditions, the complement system is constantly activated at a low-level (through the AP) and the harmful effects are prevented by various endogenous soluble and membrane-bound inhibitory molecules. For example, the MAC can be turned into harmless soluble SC5b-9 complex by S-protein/Vitronectin under physiological conditions (Su, 1996). In addition, recent evidence suggests that sub-lytic MAC may have immune modulatory roles, e.g., inducing IL-6, IL-8, CCL2 and VEGF production (Lakkaraju et al., 2014, Lueck et al., 2011). The key difference between different pathways rests on how the enzymes, i.e. C3 and C5 convertase, are formed. The convertases of C3 and C5 of the CP and the lectin pathway comprise the complement components C4bC2b and C4bC2bC3b respectively, while in the AP they are composed of C3bBb (C3 convertase) and C3bBbC3b (C5 convertase) (Fig. 2) (Zipfel and Skerka, 2009). In addition, complement can also be activated by a pathway that acts independently of C3 to bypass the C3 convertase and is mediated by direct thrombin action on the C5 convertase (Huber-Lang et al., 2006).

Traditionally the complement system is thought to be important for the elimination of invasive microbes (through cell lysis by MAC) and removal of waste (through opsonisation and promoting phagocytosis by C3b). Compelling evidence suggests that complement also plays critical roles in regulating inflammatory and immunological processes. Complement fragments C3a and C5a are anaphylatoxins and can induce vasodilation, increase the permeability of small blood vessels and induce contraction of smooth muscles (Fig. 2). C3a and C5a are chemotactic to neutrophils, mast cells, and lymphocytes. They can trigger oxidative burst in macrophages and eosinophils, and induce the release of histamine from basophils and mast cells (Fig. 2). C3a and C5a can enhance antigen presentation by DCs and promote Th1 cell differentiation (Sacks, 2010). In addition to their proinflammatory properties, C3a and C5a also participate in tissue regeneration and fibrosis (Hillebrandt et al., 2005, Strey et al., 2003).

C3a and C5a exert most of their biological activities through ligation of three cognate receptors: the C3a receptor (C3aR), the C5a receptor (C5aR), and the chemoattractant receptor-like protein C5L2. These receptors are widely expressed by immune cells and non-immune cells. Therefore, a fully activated complement pathway can modulate the immune response at multiple levels.

Recent evidence suggests that C3a can also be generated independent of complement activation through intracellular cleavage of C3 by cathepsin L (Liszewski et al., 2013). This local generation of anaphylatoxins is important for their pleiotropic biologic effects beyond inflammation.

## 5 Complement regulation in the retina

Compelling evidence suggests that a complement regulatory system exists in the retina-RPE/choroid. The mRNA of complement components including C1qb, C1r, C2, C3, C4, complement factor B (CFB), and factor H (CFH) have been detected in the retina and RPE/choroid of human (Anderson et al., 2010) and mouse eyes (Luo et al., 2011). Retinal microglia and RPE cells appear to be the major cellular sources of local complement expression (Luo et al., 2011). The retina/RPE/choroid also expresses complement regulatory proteins. For example, CD46 was detected at the basal surface of RPE cells (Fett et al., 2012, Vogt et al., 2006), CD55 in ganglion and photoreceptors, CD59 in retinal nerve fibers (Vogt et al., 2006), and CFH in RPE (Chen et al., 2007) and choriocapillaris (Fett et al., 2012). The complement receptor CR1 and C3aR were detected in ganglion cells (Fett et al., 2012), whereas C5aR was detected in the inner plexiform layer (Vogt et al., 2006). In vitro studies have shown that RPE cells (Fukuoka and Medof, 2001), astrocytes (Gasque et al., 1995), and Muller cells (Cheng et al., 2013) all express C5aR.

An age-dependent upregulation of complement genes was observed in the retina (Chen et al., 2010a) and RPE/choroid (Chen et al., 2008a, Chen et al., 2008b) in mice. A recent study has reported an age-related accumulation of MAC in the choriocapillaris of healthy donor eyes (Chirco et al., 2015, Mullins et al., 2014). We have previously shown that expression of complement C3, C4, and CFB in mouse retina can be affected by cataract surgery (Xu et al., 2011) and irradiation (Chen et al., 2012). In vitro studies have further shown that complement expression by retinal cells is regulated by inflammatory cytokines and chemokines. For example, the expression of complement and regulatory genes by RPE cells can be regulated by cytokines such as TNF-α, IFN-γ, IL-27 (Amadi-Obi et al., 2012, Chen et al., 2007, Chen et al., 2008b, Lau et al., 2011) or the supernatants of macrophages (Luo et al., 2013). These results suggest that the retinal complement system is actively responding to ocular microenvironmental stimulations.

Although the physiological role of the complement system in retinal health and disease is not fully understood, the fact that the retina only express selected components of complement proteins and regulatory molecules suggests that the complement system cannot fully be activated if the BRB is intact. It also suggests that the retina has the capacity to control local complement activation when the BRB is broken down. This may have important implications in developing complement based therapy for retinal diseases, i.e., controlling complement activation locally in the retina rather than systemically may be a more effective approach.

## 6 Complement activation and retinal diseases

### 6.1 Complement activation in Uveoretinitis

Uveoretinitis is an inflammatory condition that involves the uveal tract and the retina of the eye. The disease can cause devastating vision loss if left untreated. Current treatment of uveoretinitis is through local or systemic administration of immunosuppressants, which is often associated with severe adverse effects such as cataract and glaucoma and there is an urgent need to develop new effective and safe therapies.

Complement activation is known to be involved in the pathogenesis of uveoretinitis. The aqueous humor of uveitis patients contains high levels of C3a, C3c and CFB (Mondino et al., 1984). Polymorphisms in complement genes, including CFH, SERPING (Thompson et al., 2013, Yang et al., 2011, Yang et al., 2013), and C5 (Xu et al., 2015) increase the risk of uveitis. EAU is an established model of human posterior uveoretinitis (Caspi et al., 1990, Caspi et al., 1994, Forrester et al., 1992). The disease represents a T cell driven autoimmune response to retinal antigens (Caspi et al., 1990, Liversidge and Forrester, 1988) in which both Th1 and Th17 cells are involved (Amadi-Obi et al., 2007, Luger et al., 2008). Mice deficient in complement C3 are less susceptible to EAU (Read et al., 2006), whereas mice deficient in the decay-accelerating factor (DAF) develop greater EAU than their wild-type controls (An et al., 2009). Furthermore, EAU can be suppressed by introducing the soluble complement inhibitor (sCrry) (Read et al., 2006), recombinant DAF (An et al., 2009), or complement C5 monoclonal antibody (Copland et al., 2010). We have shown that CFB mRNA was significantly increased in EAU retina and that blocking the AP complement activation using fusion protein CRIg-Fc could suppress retinal inflammation in the mouse model of EAU (Chen et al., 2010b).

The complement system may contribute to retinal pathology in uveoretinitis at multiple levels. In addition to MAC-mediated cell death, C3a and C5a may modulate T cell activation. C5a/C5aR pathway is known to be involved in Th1 cell activation and deletion this pathway leads to Th17 and T regulatory (Treg) cell differentiation (Weaver et al., 2010, Xu et al., 2010). Mice deficient in C3aR/C5aR are resistant to EAU induction (Zhang et al., 2015).

### 6.2 Complement activation in AMD

AMD is the progressive degeneration of the macula (central part of the retina) in people aged over 55 years. AMD is the leading cause of blindness in the elderly in developed countries, accounting for 8.7% of all blindness worldwide and is predicted to affect 196 million people by 2020 (Wong et al., 2014).

The early stages of the disease is characterised by large drusen (>63 µm) and hypo- and hyper-pigmentation of RPE in the macula (Ferris et al., 2013). The disease may progress into two late stages, dry- and wet-AMD. Wet AMD (also called neovascular AMD, nAMD) is a condition in which abnormal blood vessels grow into the subretinal space of the macula causing visual damage. Wet AMD accounts for two thirds of all late stage AMD. The disease is treated by intravitreal injection of VEGF inhibitors (e.g., Avastin, Lucentis®, Eyelea) (Heier et al., 2006, Holz et al., 2014, Kodjikian et al., 2014). Dry AMD (also called geographic atrophic, GA) is caused by RPE cell death and photoreceptor degeneration, for which there is currently no treatment.

The role of the complement system in the pathogenesis of AMD has been studied extensively over the past decade and a few excellent review articles have detailed the advancements in this field (Bora et al., 2015, McHarg et al., 2015, Warwick et al., 2014). Here we summarize the key evidence supporting the role of the complement system in the pathogenesis of AMD. Firstly, various complement components, including C3, C5b-9, CFB, and CFH have been detected in drusen as well as in AMD lesions (Anderson et al., 2002, Anderson et al., 2010). In addition, increased plasma levels of C3a, C3d, Bb, and C5a have been observed in AMD patients (Reynolds et al., 2009, Scholl et al., 2008, Lechner et al., 2016). These results suggest increased local and systemic complement activation in AMD. Secondly, polymorphisms in a number of complement genes (CFH, CFB, C2, SERPING1, and C3) increase the risk of AMD (Cipriani et al., 2012, Edwards, 2008, Katta et al., 2009). The genetic evidence suggests that the complement system, and in particular the alternative pathway may be dysregulated in AMD patients. Last but not least, experimental studies have shown that inhibition of complement activation via either systemic or local routes can suppress laser-induced CNV. Inhibition of C3a, C5a (Nozaki et al., 2006), CFB, and MAC (Lipo et al., 2013), or administration of the complement regulatory molecules CD59 (Bora et al., 2010) and CFH (Kim et al., 2013) can suppress CNV development in animal models.

### 6.3 Complement activation in DR

DR is a progressive degeneration of retinal vasculature and neurons as a result of diabetes. The longer a person has diabetes, the higher the chance he/she might develop DR. After 20 years of the disease, nearly all patients with type 1 diabetes will have at least some DR. For type 2 diabetes, around 80% who are insulin-dependent and 50% who are non-insulin-dependent will have DR after 20 years (Romero-Aroca et al., 2010). DR is the leading cause of blindness among people of working age in Western countries (Aiello et al., 1998).

In the early stages, patients may present with microaneurysms, hard exudates, haemorrhages, and cotton-wool spots in the fundus. The diseased vessels may leak fluid from the circulation into the macula (i.e. diabetic macular oedema) leading to severe vision loss. As the disease progresses, new blood vessels may grow (proliferative DR) as a result of severe isehemia. The new blood vessels are fragile and can cause severe haemorrhage and ultimately destroy the retina.

In addition to vascular damage, retinal neural cells may also be affected even at the early stages of the diabetes. There is growing evidence to suggest that retinal neuron damage, in particular the reactive oxygen species (ROS) released by stressed photoreceptors is an early event in DR pathogenesis (Du et al., 2013, Simo et al., 2012).

The underlying mechanisms leading to retinal vasculopathy and neuropathy in diabetes are not fully understood, although oxidative stress and inflammation are known to be important detrimental factors. The role of inflammation and various inflammatory mediators (such as cyclooxygenase (COX), TNF-α, IL-1β, and HMGB-1), AGEs, and S100B in different stages of DR pathologies has been reviewed extensively elsewhere (Chen et al., 2013a, Chen et al., 2013b, Tang and Kern, 2011). However the role of the complement system in DR pathogenesis is less well appreciated.

Early work by Gerl et al. (2002) demonstrated that choriocapillaris of DR eyes contain significant levels of C3d and the C5b-9 complex. C5b-9 deposition was also detectable in retinal vessels of patients with >9-year T2D (Zhang et al., 2002). Increased C5a was detected in the vitreous of patients with proliferative DR (Muramatsu et al., 2013). Muller cells constitutively express C5aR and the expression can be upregulated by hyperglycemia and inflammatory stimuli such as PGE2 (Cheng et al., 2013). The ligation of C5aR with C5a in Muller cells results in the release of IL-6 and VEGF (Cheng et al., 2013); both are known to be critically involved in DR pathology. The results suggest that complement activation is involved in retinal vascular damage in DR (Nozaki et al., 2006).

How the complement system is activated in the diabetic eye is not known. The expression of complement inhibitors CD55 and CD59 was reduced in retinal vessels of DR eyes (Zhang et al., 2002). Interestingly, C1q, C4 and MBL were not detected in the DR eyes (Gerl et al., 2002, Zhang et al., 2002), indicating that the complement system may be activated through the alternative pathway in DR.

### 6.4 Complement activation in glaucoma

Primary glaucoma is an age-related chronic optic neuropathy resulting from increased intraocular pressure (IOP). Glaucoma causes progressive peripheral vision loss and, if untreated, may lead to blindness. The most representative pathologic finding in glaucoma is the death of retinal ganglion cells by apoptosis.

Current treatment for glaucoma is aimed at reducing IOP using medicinal or surgical approaches. However a significant number of patients continue to lose vision after IOP is controlled. Compelling evidence suggests that inflammation, including complement activation contributes to ganglion cell death in glaucoma. Tezel et al. (2010) reported the upregulation of various complement components (predominately involved in CP) and down-regulation of complement inhibitors in glaucoma retinae using proteomic analysis. Using microarray techniques, Howell et al. (2011) showed that the complement cascade was upregulated at the early stages of glaucoma in DBA/2J mice. In the experimental model of glaucoma, C5b-9 deposition was detected at the ganglion cell layer and complement depletion reduced ganglion cell loss (Jha et al., 2011). Furthermore, mice with C1qa or C5 (Howell et al., 2011, Howell et al., 2013) deficiency were protected from glaucoma. These results suggest that complement activation through the classical pathway may contribute to retinal ganglion cell death in glaucoma.

## 7 Targeting the complement system for the management of retinal disease

Despite convincing evidence that complement activation contributes to retinal pathology in the aforementioned sight-threatening diseases, so far the clinical predictive or therapeutic value of these findings has not materialized. When targeting the complement system for therapy, it is important to evaluate the beneficial and detrimental aspects of complement activation in retinal disease. On one hand, the generation of C3b is beneficial for resolution of inflammation and tissue remodelling (through promoting phagocytosis of dead cells and debris). On the other hand, MAC-mediated cell death or C3a/C5a induced immune activation may contribute to retinal pathology. Ideally the therapy should be sufficient to constrain exacerbated complement activation without shutting down necessary biological functions pertinent to immune surveillance or tissue homeostasis. Complement activation involves a cascade of multiple steps, and each step might be targeted for therapy. The chosen therapy should consider the pathologic mechanisms of the disease such as the triggers of complement activation, the pathways, the location, and the disease course.

### 7.1 General principles for developing complement-targeted drugs

*Avoiding toxicity*: The complement system has many essential physiological roles in retinal defences. Therefore, any complement-targeted therapy should cause as little disruption of its physiological roles as possible, particularly if the treatment is to be continued in the long-term. The strategies to reduce the adverse effects related to complement inhibition may include: (1) targeting down-stream of the complement pathway, i.e., MAC formation, for which the only risk is infection; (2) transient inhibition of complement activation (may only be applicable for acute diseases); (3) targeting complement activation locally in the retina. This should be the preferred approach to manage retinal diseases that are caused predominately by local variants, such as glaucoma and AMD. However, both glaucoma and AMD are chronic diseases, and may require long-term (years) management. The toxicity of long-term complement inhibition in the retina should be carefully considered. In DR and uveoretinitis the ocular barrier is often disrupted and systemic immune alteration contributes significantly to retinal pathogenesis. Although control of local immune activation, including complement activation is important for preserving retinal structure and function, rectifying the systemic immune response should also be considered, particularly at the acute stage of the disease.

*Selecting targets for complement inhibition:* How to control the complement system for the management of retinal diseases is an important and but often difficult question. With various potential sites of inhibition to be considered, the knowledge of how the complement system is activated and which pathways are involved will be essential to identify the target of inhibition. In general, the targets that can be considered for therapy may include the initiators, regulators, and amplification of the cascade, and the effectors of the complement system. The initiators include complement components that form the activation complex (e.g., C1q in the CP, C3 in the AP, and MBL in the lectin pathway) and relevant enzymes (C1r and C1s in the CP, MASP1 and MASP2 in the lectin pathway, and C3 convertases in the AP). The regulators include membrane-bound molecules (SIGNR, CR1-4, CRIg, CD46, CD55, CD59) and soluble proteins (C4BP, C1INH, CFH, CFHR1, FHL1, properdin, clusterin, vitronectin) that negatively regulate complement activation (Zipfel and Skerka, 2009) as well as enzymes involved in the amplification stage (e.g., factor D). The effectors include C5b-9 (MAC) and the anaphylactic proteins C3a and C5a. The targeting approaches may include neutralizing antibodies against the assembling compounds, enzymes or the synthetics of the inhibitors. There are a number of complement inhibitors that selectively target each of the above points that have been approved by the United States Food and Drug Administration (FDA) or the European Medicines Agency (EMA) in clinical development and others are in pre-clinical development for inflammatory diseases such as hemolytic uremic syndrome, ischemia-reperfusion injury, organ transplant rejection, and thrombotic microangiopathy (Reis et al., 2015) (Table 1).

In different retinal diseases the complement system contributes differently to the pathogenesis. Therefore, treatment strategies should differ depending on disease pathogenesis. In uveoretinitis, for example, the effectors (MAC, C5a) are known to play a key role in retinal damage, and a previous study has shown that targeting C5 (local or systemic) could effectively suppress experimental autoimmune uveoretinitis (Copland et al., 2010). Regrettably, little is known about how the complement system damages the retina in other disease conditions including AMD despite many years of intensive study. That is reflected by the poor outcomes in complement based clinical trials (see below).

### 7.2 Current progress in complement based therapy for retinal diseases

There are a number of complement-targeting drugs in clinical (Table 2) and pre-clinical trials for retinal diseases, predominately for the management of AMD with only one trial for non-infections uveitis patients (NCT01526889, Table 2), and two trials for optic neuritis (NCT02003144, NCT01759602, Table 2).

#### 7.2.1 Non-infectious Uveitis

LFG316 is a C5-specific mAb developed by Novartis. It is in phase 2 clinical trials for a number of complement related diseases, including Paroxysmal nocturnal hemoglobinuria (PNH), AMD, and non-infectious uveitis. In the uveitis study (NCT01526889), LFG316 will be administered intravitreally (IVT) to patients with active, non-infectious intermediate-, posterior-, or panuveitis, and the safety, efficacy and pharmacokinetics will be assessed over a 12-week period. The trial is ongoing.

#### 7.2.2 AMD

There are a number complement inhibitors are in phase 1-3 clinical trials for the treatment of AMD (Table 2), including one inhibitor (POT-4) targeting C3, four (LFG316, Eculizumab, Zimura, and ARC1905) targeting C5, and another one targeting factor D (Lampalizumab formerly FCFD4514S). The majority of the trials have shown a good record of safety, however the efficacy remains to be determined.

**C3-targeted inhibitor:** POT-4/Compostatin is a cyclic peptide that inhibits C3 cleavage and prevents complement activation. An in vitro study has shown that compostatin could inhibit drusen-like deposits in human RPE cultures (Gorham et al., 2013). Furthermore, an in vivo study in a monkey model of early-onset macular degeneration observed the disappearance of drusen 6 months after intravitreal injection of compostatin (Chi et al., 2010). A phase 1 study to evaluate the safety and tolerability of intravitreal POT-4 injection for treatment of patients with neovascular AMD was conducted between 2007 and 2010 (NCT00473928). However, positive results, if any, have never been reported.

**C5-targeted inhibitors:** LFG316 is a fully-human, high-affinity antibody against C5 and can prevent C5 cleavage. The phase 2 studies of LFG316 for both GA (NCT01527500) and neovascular AMD (NCT01535950) were completed in 2015, and the results are yet to be reported.

Eculizumab, another C5 specific mAb, had been approved for the treatment of PNH and atypical hemolytic uremic syndrome (aHUS) (Rother et al., 2007). A phase 2 study has shown that up to 24 weeks intravenous infusions of Eculizumab was well-tolerated by AMD patients through 52 weeks follow-up (Yehoshua et al., 2014). However, neither GA progression (Yehoshua et al., 2014) nor drusen volume (Garcia Filho et al., 2014) was affected by the therapy despite significant reduction on circulating C5 activity (to less than 9% of normal levels by week one and less than 1% by week two after treatment) (Yehoshua et al., 2014). The results suggest that systemic complement activation may have limited effect on AMD pathology. Further study on the effect of intravitreal administration of Eculizumab in AMD has not been reported.

Zimura® and ARC1905 are aptamer C5 inhibitors developed by the Ophthotech Corporation. Zimura® is in phase 2 trials as a combined therapy with anti-VEGF for the management of neovascular AMD (NCT02397954), whereas ARC1905 is being tested for both GA (NCT00950638) and nAMD (NCT00709527).

**CFD-targeted inhibitor:** Factor D (CFD) is responsible for cleaving factor B, an essential substrate for the formation of C3 convertase (C3bBbC3b) of the AP. Targeting CFD can effectively suppress AP-mediated complement activation. Lampalizumab (FCFD4514S) is a humanized IgG Fab fragment directed against CFD. Intravitreal injection of lampalizumab in cynomolgus monkeys at high doses (>10 mg) resulted in transient (0.5–3 h) inhibition of systemic AP activity (Loyet et al., 2014). The results from phase 1–2 studies were published on June 11, 2015 in Clinical Ophthalmology (Rhoades et al., 2015). However, the paper was retracted two weeks later (June 26, 2015) because “the results referenced in this manuscript are preliminary analyses and may not reflect the final data and conclusions of the clinical trials cited”. Nevertheless, the paper reported that (1) a phase 1 study showed that intravitreal injection of lampalizumab (up to 10 mg) was well-tolerated through 90 days by GA patients; (2) a phase 2 study (MAHALO) showed that the size of GA reduced by 20.4% from baseline after 18 months treatment. Further subgroup analysis revealed a 44% reduction in patients who were positive for exploratory biomarkers (i.e., mutations for CFH, C3, or C2/CFB) (P<0.005) (Rhoades et al., 2015). These results were originally reported by Holz et al at the 14th EURETINA Congress in 2014 (http://www.euretina.org/london2014/programme/free-papers-details.asp?id=3439&day=0). Phase 3 clinical trials to evaluate the efficacy and safety of intravitreal injection of lampalizumab for the treatment of GA are ongoing (Table 2) (PharmaTimes).

Despite millions of dollars having been spent and nearly a decade of intense research, the results so far from the clinical trials have produced disappointing primary outcomes (i.e., minimal improvement in visual acuity or reduction in disease progression) (Table 2). Most of these trials have ended early as a result of disappointing interim results and others with no reason reported (with only one exception, Table 2). The disappointing results from clinical trials have highlighted our lack of basic understanding about the mechanisms by which complement factors influence AMD development (Reynolds et al., 2009, Scholl et al., 2008). Thus we are unable to address precisely when, where, and how to inhibit complement activation in different types of AMD.

Since AMD has strong genetic predisposition, it has been proposed that genetic screening may help to predict treatment responsiveness. Indeed the MAHALO study, although preliminary, has observed better responses to Lampalizumab from GA patients with complement gene mutations (Rhoades et al., 2015). However, the data about whether polymorphisms in complement genes and other AMD risk factor genes can be used to predict the response to VEGFA‑targeted therapies are conflicting (Hagstrom et al., 2013, Smailhodzic et al., 2012). Similarly, genetic models fail to predict progression to geographic atrophy (Klein et al., 2010, Scholl et al., 2009). We have shown recently that the level of complement activation differs in different types of AMD (Lechner et al., 2016). A complex approach, taking into account genetic information, environmental risk factors, biological phenotypes and clinical presentation, may be a more appropriate way forward in developing complement based personalized therapy for AMD and other retinal diseases.

## 8 Future perspectives and conclusions

The discovery of the link between complement gene polymorphisms and AMD risk has prompted a resurgence in investigating the role of the complement system in retinal diseases. As a result, our knowledge of this topic has advanced considerably in the past decade, which has prompted the design of distinct strategies to develop complement based therapies for AMD patients. There are a few FDA approved complement inhibitors for other inflammatory diseases, and clinical experience from these studies suggests that complement-targeted therapies can be safe and effective. In addition, there are over 40 ongoing clinical trials evaluating different classes of complement inhibitors in numerous diseases. These clinical studies will provide valuable information about the safety and efficacy of distinct strategies for complement targeting, which offers a great opportunity to develop complement-based therapies for sight-threatening retinal diseases. For each retinal disease, future research should address how the complement system is activated, which complement pathway is involved, which complement components cause retinal pathology and at which stage of the disease, and whether the complement system affects the eye systemically or locally. This knowledge will be important in deciding whether complement inhibition should be applied systemically or locally to treat disease, and when and how the complement system should be targeted for therapy. As complement activation is not the initial cause of the disease, it is understandable that complement inhibition on its own may not be sufficient to control retinal pathology, and concomitant targeting of disease aetiology may maximize the benefits.

## Disclosures

The authors declare no competing financial interests.

## Figures and Tables

**Table 1:** Complement inhibitors that have been approved or are in preclinical or clinical studies.

**Target point**	**Compounds**	**Mechanism of inhibition**	**Company**	**Application and Status**
**Initiators**	Berinert	Plasma-derived human C1-INH	CSL Behring	Approved for hereditary angioedema (HAE)
	Ruconest	C1 esterase inhibitor (recombinant)	Pharming Group N.V.	Approved for HAE
	Cinryze	Plasma-derived human C1-INH	ViroPharma Biologics	Approved for HAE
	TNT009	C1 s inhibitor (mAb)	True North Therapeutics	Phase 1
	OMS721	MASP-2 antibody	Omeros Corporation	Thrombotic microangiopathies, phase 2
**Regulators or amplification cascades**	Lampalizumab	Factor D mAb fragment	Genentech	Phase 3 trials for AMD
	APL-1/2	C3 (Compstatin derivatives)	Apellis Pharmaceuticals	Phase 1 for Paroxysmal nocturnal hemoglobinuria (PNH)
	AMY-101	C3 (Compstatin derivatives)	Amyndas Pharmaceuticals	Pre-clinical study in PNH
	TP10	Soluble CR1	Celldex	Phase 1 for patients with C3 glomerulopathy
				Phase 2 for adult women undergoing cardiopulmonary bypass surgery
	TT30	Fusion protein linking CR2 and CFH	Alexion Pharmaceuticals	Phase 2 or PNH
	Mini-FH	Engineered version of CFH	Amyndas Pharmaceuticals	Pre-clinical study in C3 glomerulopathy
**Effectors**	Mubodina/Ergidina	C5 specific mAb	Adienne S.A.	Pre-clinical study in aHUS
	Eculizumab	C5 specific mAb	Alexion Pharmaceuticals	Approved for PNH and aHUS; Phase 1–2trials for neuromyelitis optica, kidney graft reperfusion injury, AMD, myasthenia gravis, Guillain-Barre syndrome, etc.
	Zimura, ARC1905	Both are synthesized C5 inhibitors (aptamer)	Ophthotech	Phase 2 for AMD
	LFG316	C5 specific mAb	Novartis	Phase 2 for AMD, PNH and non-infectious uveitis
	Coversin	Recombinant small protein (prevents C5 cleavage)	AKARI Therapeutics	Phase 2 for PNH
	ALN–CC5	C5 specific RNAi	Alnylam	Phase 1 and 2 for PNH
	CaCP29	C5a specific mAb	InflaRx	Phase 2 for septic organ dysfunction
	CCX168	C5aR inhibitor	ChemoCentryx	Phase 2 for aHUS, ANCA-associated vasculitis, and IgA nephropathy

**Table 2:** Complement based clinical trials for retinal diseases

**Compound**	**Target**	**Structure**	**Indications**	**Trial status**	**Trial number**
**POT-4/Compstatin (Alcon)**	C3	Cyclic peptide inhibitor	Neovascular AMD	Phase 1 ended in 2010 (data, unpublished)	NCT00473928
**LFG316 (Novartis)**	C5	Monoclonal antibody	Geographic atrophy	Phase 2 for GA, completed in 2015 (Data, unpublished)	NCT01527500
			Advanced neovascular AMD	Phase 2 for wet AMD, completed in 2015 (Data, unpublished)	NCT01535950
			Non-infectious uveitis	Phase 2, ongoing	NCT01526889
**Eculizumab (Alexion Pharmaceuticals)**	C5	Monoclonal antibody	Dry AMD	Phase 2 completed (Yehoshua et al., 2014; Garcia Filho et al., 2014)	NCT00935883
			Optic Neuritis, myelitis,	Phase 3, ongoing	NCT02003144
**Zimura® (Ophthotech)**	C5	Aptamer	Neovascualr AMD in combination with anti-VEGF	Phase 2 ongoing	NCT02397954
**ARC1905 (Ophthotech)**	C5	Aptamer	Neovascular AMD	Phase 1 ended in 2012 (data, unpublished)	NCT00709527
			Geographic atrophy	Phase 1 ended in 2013 (data, unpublished)	NCT00950638
**Lampalizumab/FCFD4514S (Genentech, Hoffmann-La Roche)**	Factor D	Monoclonal antibody (Fab fragment)	Geographic atrophy	Phase 1 completed (Rhoads et al. 2015)	NCT00973011
				Phase 2, ongoing	NCT01602120
					NCT01229215
					NCT02288559
				Phase 3, ongoing	NCT02247479
					NCT02247531
**CINRYZE® (Michael Levy)**	C1-INH	C1 esterase inhibitor	Neuromyelitis optica (NMO), optic neuritis	Phase 1, completed (Levy and Mealy, 2014)	NCT01759602
